# Deletion of TAK1 in the Myeloid Lineage Results in the Spontaneous Development of Myelomonocytic Leukemia in Mice

**DOI:** 10.1371/journal.pone.0051228

**Published:** 2012-12-10

**Authors:** Betty Lamothe, YunJu Lai, Lana Hur, Natalia Martin Orozco, Jing Wang, Alejandro D. Campos, Min Xie, Michael D. Schneider, Cynthia R. Lockworth, Jared Jakacky, Diep Tran, Michael Ho, Sity Dawud, Chen Dong, Hui-Kuan Lin, Peter Hu, Zeev Estrov, Carlos E. Bueso-Ramos, Bryant G. Darnay

**Affiliations:** 1 Department of Experimental Therapeutics, The University of Texas MD Anderson Cancer Center, Houston, Texas, United States of America; 2 Department of Immunology, The University of Texas MD Anderson Cancer Center, Houston, Texas, United States of America; 3 Department of Molecular and Cellular Oncology, The University of Texas MD Anderson Cancer Center, Houston, Texas, United States of America; 4 Department of Internal Medicine, The University of Texas Southwestern Medical Center, Dallas, Texas, United States of America; 5 Faculty of Medicine, National Heart and Lung Institute, Imperial College London, London, United Kingdom; 6 Department of Veterinary Medicine and Surgery, The University of Texas MD Anderson Cancer Center, Houston, Texas, United States of America; 7 School of Health Professions, The University of Texas MD Anderson Cancer Center, Houston, Texas, United States of America; 8 Department of Leukemia, The University of Texas MD Anderson Cancer Center, Houston, Texas, United States of America; 9 Department of Hematopathology, The University of Texas MD Anderson Cancer Center, Houston, Texas, United States of America; Cincinnati Children's Hospital Medical Center, United States of America

## Abstract

Previous studies of the conditional ablation of TGF-β activated kinase 1 (TAK1) in mice indicate that TAK1 has an obligatory role in the survival and/or development of hematopoietic stem cells, B cells, T cells, hepatocytes, intestinal epithelial cells, keratinocytes, and various tissues, primarily because of these cells’ increased apoptotic sensitivity, and have implicated TAK1 as a critical regulator of the NF-κB and stress kinase pathways and thus a key intermediary in cellular survival. Contrary to this understanding of TAK1’s role, we report a mouse model in which TAK1 deletion in the myeloid compartment that evoked a clonal myelomonocytic cell expansion, splenomegaly, multi-organ infiltration, genomic instability, and aggressive, fatal myelomonocytic leukemia. Unlike in previous reports, simultaneous deletion of TNF receptor 1 (TNFR1) failed to rescue this severe phenotype. We found that the features of the disease in our mouse model resemble those of human chronic myelomonocytic leukemia (CMML) in its transformation to acute myeloid leukemia (AML). Consequently, we found TAK1 deletion in 13 of 30 AML patients (43%), thus providing direct genetic evidence of TAK1’s role in leukemogenesis.

## Introduction

Transforming growth factor β-activated kinase 1 (TAK1; MAP3K7), a member of the mitogen-activated protein kinase kinase kinase (MAP3K) family, was initially identified as a kinase in TGFβ signaling [1]. However, recent *in vitro* studies have revealed that TAK1 is involved in the cytokine-mediated activation of the IκB kinase (IKK)/nuclear factor κB (NF-κB) and stress kinase (*i.e.*, p38 and c-Jun N-terminal kinase [JNK]) pathways via a number of inflammatory and immune regulators such as tumor necrosis factor α (TNFα), interleukin-1β (IL1-β), and Toll-like receptor ligands and T- and B-cell receptor engagement [2], [3]. Thus, TAK1 is a central player in the regulation of a diverse array of cellular processes.

The offspring of TAK1-floxed mice crossed with mice expressing cell- or tissue-specific Cre recombinase have been used to investigate the physiological roles of TAK1 in various cell types. Conditional ablation of TAK1 in epidermal [4], [5] or intestinal epithelial cells [6] revealed that TAK1 has a functional role in preventing keratinocyte death and skin inflammation as well as enterocyte apoptosis and inflammatory bowel disease. Although these mice died postnatally, the simultaneous deletion of TNF receptor 1 (TNFR1) could prevent some of these phenotypic characteristics [4]–[6]. In addition, the hepatocyte-specific deletion of TAK1 in mice resulted in spontaneous hepatocyte death, inflammation, and fibrosis, which was partially prevented in a TNFR1-null background [7], leading to compensatory proliferation and fatal liver cancer via an NF-κB-independent pathway [8]. Cartilage-specific deletion of TAK1 resulted in defective chondrocyte maturation and joint development, and postnatal death [9], [10], whereas deletion of TAK1 in osteoblasts led to features similar to the human disease cleidocranial dysplasia [11].

Within the hematopoietic compartment, conditional knockout of TAK1 in T cells showed dramatic defects in thymocyte and regulatory T cell development and signaling [12]–[14] whereas TAK1-deficient B cells showed defects in signaling by Toll-like receptor ligands, CD40, and B-cell receptor [15], [16]. Inducible deletion of TAK1 using the Mx1-Cre mouse model [17] caused complete bone marrow failure and massive liver failure in 8–10 days, which was due to cell autonomous apoptotic death of all hematopoietic cells and hepatocytes [17] that could be partially rescued by simultaneous deletion of TNFR1 and TNFR2 [18]. Furthermore, TAK1 deletion in the myeloid lineage increased cytokine production in response to lipopolysaccharide (LPS), both *in vivo* and *in vitro* in 6- to 12-week-old mice [19], [20], although conflicting data was reported on the role of TAK1 in LPS signaling *in vitro*. Thus, TAK1 is a crucial component for maintaining homeostasis, survival, and differentiation and preventing spontaneous apoptosis in a number of different mouse models.

Even though two studies highlighted a critical role of TAK1 in the myeloid lineage following endotoxin challenge [19], [20], further analysis of age-related diseases associated with TAK1 deficiency in the myeloid lineage was not elucidated. Several years ago, we generated a myeloid-specific knockout of TAK1 in mice to investigate the role of TAK1 in osteoclast differentiation. Surprisingly, we found that these mice had significantly larger spleens than their littermates did and succumbed to an early death. In the present study, we sought to investigate this unexpected phenotype in greater detail. We found that these mice developed myelomonocytic cell expansion, splenomegaly, multi-organ infiltration, genomic instability, and aggressive, fatal myelomonocytic leukemia with complete penetrance. These mice exhibited features similar to those of humans with chronic myelomonocytic leukemia (CMML) in transformation to acute myeloid leukemia (AML). Analysis of bone marrow samples from patients with CMML or AML revealed that the deletion of TAK1 in leukemic blast cells is associated with CMML in transformation to AML or acute myelomonocytic leukemia (AMML). These findings suggest that defective TAK1 has a causative role in the clonal proliferation of myelomonocytic cells that characterizes CMML.

**Figure 1:**
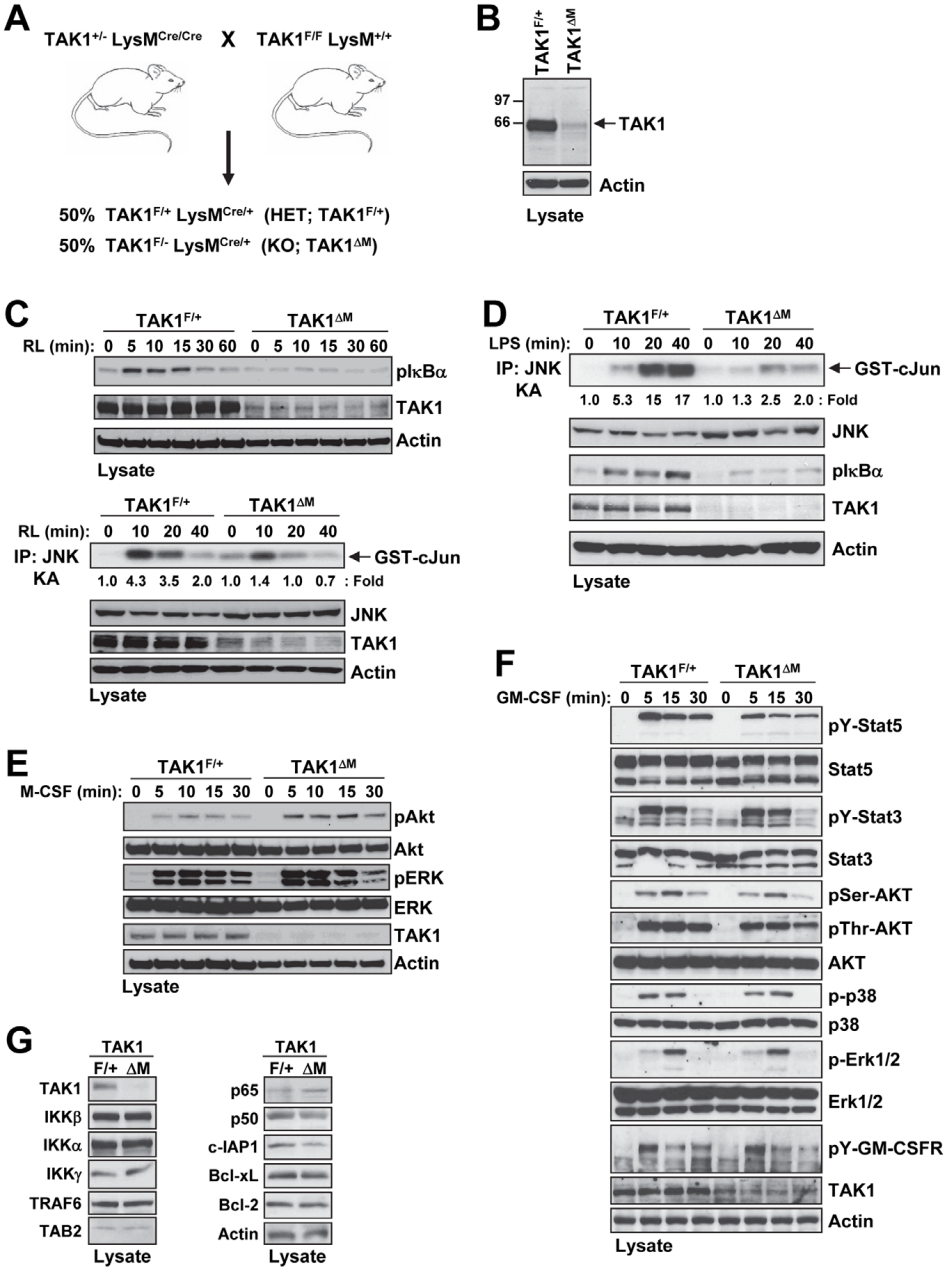
Myeloid-specific deletion of TAK1 prevents RANKL and LPS signaling, but not M-CSF and GM-CSF signaling. (**A**) The breeding scheme used to generate mice with TAK1 deleted in the myeloid lineage. (**B**) Western blot analysis revealed TAK1 deletion in CD11b^+^ cells. Bone marrow cells from the indicated mice were harvested, and CD11b^+^ cells were sorted by magnetic beads. Cell lysates were subjected to SDS-PAGE and immunoblotted with the indicated antibodies. (**C–D**) Primary BMMs from TAK1^F/+^ and TAK1^ΔM^ mice were treated with RANKL (200 ng/ml) (**C**) or LPS (200 ng/ml) (**D**) for the indicated times. Cell lysates were subjected to SDS-PAGE and immunoblotted with the indicated antibodies. In addition, cell lysates were immunoprecipitated with anti-JNK1, and an *in vitro* kinase assay (KA) was performed. (**E**) Primary BMMs from TAK1^F/+^ and TAK1^ΔM^ mice were treated with M-CSF (10 ng/ml) for the indicated times. Cell lysates were subjected to SDS-PAGE and immunoblotted with the indicated antibodies. (**F**) BM cells from TAK1^F/+^ and TAK1^ΔM^ mice were isolated, and CD11b^+^ cells were sorted by magnetic beads and treated with GM-CSF (20 ng/ml) for the indicated times. Cell lysates were subjected to SDS-PAGE and immunoblotted with the indicated antibodies. (**G**) Cell lysates from primary BMMs from TAK1^F/+^ and TAK1^ΔM^ mice were subjected to SDS-PAGE and immunoblotted with the indicated antibodies.

## Materials and Methods

### Ethical Considerations

All mouse experiments were approved by The University of Texas MD Anderson Cancer Center’s Institutional Animal Care and Use Committee (protocol #: 09-06-12932). Mice were cared for in accordance with guidelines set forth by the American Association for Accreditation of Laboratory Animal Care and the U.S. Public Health Service Policy on Humane Care and Use of Laboratory Animals.

**Figure 2:**
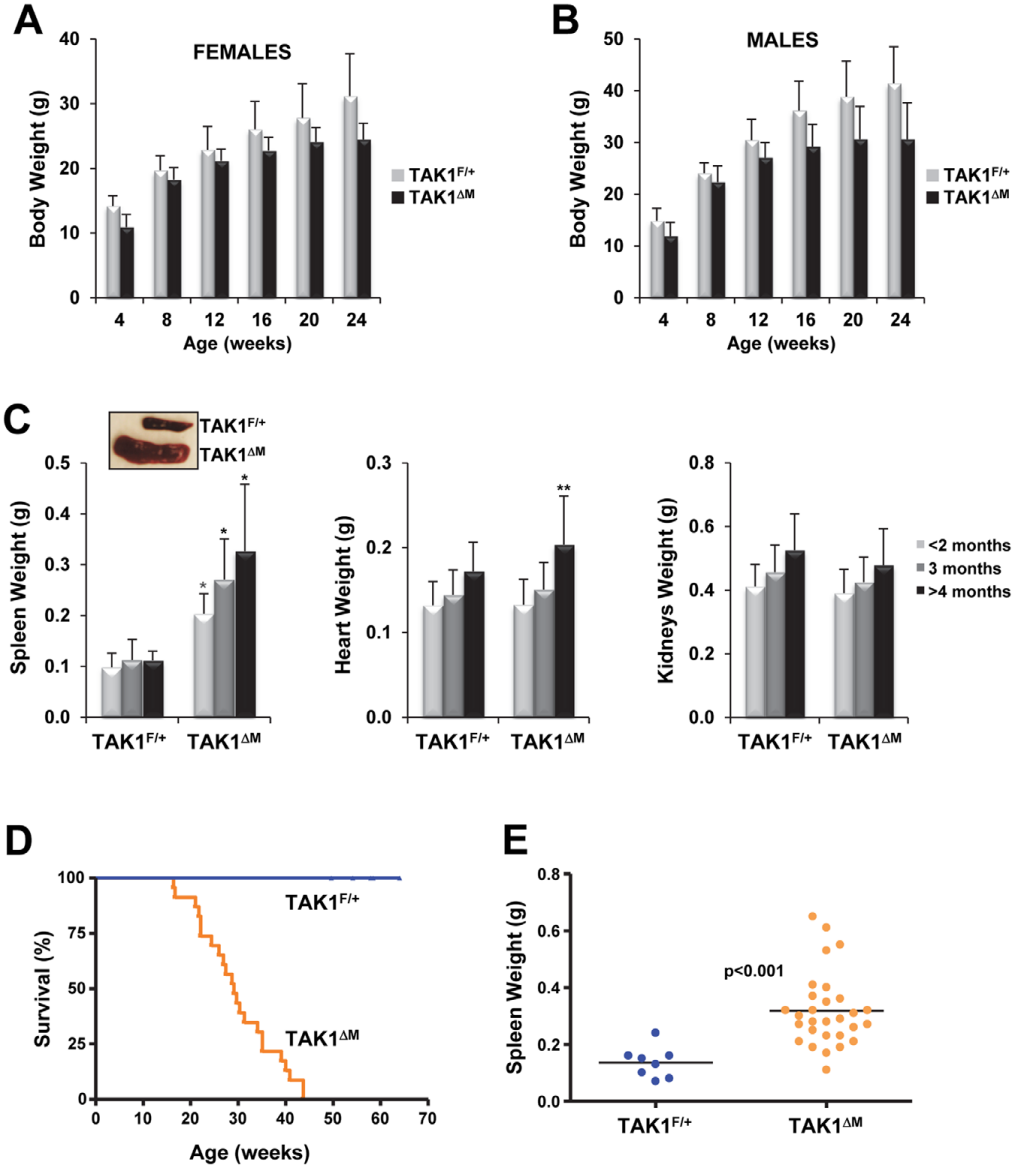
TAK1^ΔM^ mice develop splenomegaly and have shortened lifespans. (**A–B**) Decreased body weights of aged TAK1^ΔM^ mice. The mean (± SEM) body weights of cohorts of female (**A**) and male (**B**) TAK1^F/+^ and TAK1^ΔM^ mice age 4–24 weeks at 4-week intervals are shown. For TAK1^F/+^ females, n = 16–36; for males, n = 16–22. For TAK1^ΔM^ females, n = 16–31; for males, n = 9–19. For females: *P*<0.0001 (4 weeks), *P* = 0.0071 (8 weeks), *P* = 0.0206 (12 weeks), *P* = 0.0003 (16 weeks), *P* = 0.0020 (20 weeks), and *P* = 0.0006 (24 weeks). For males: *P* = 0.0005 (4 weeks), *P* = 0.0637 (8 weeks), *P* = 0.0169 (12 weeks), P = 0.0004 (16 weeks), *P* = 0.0017 (20 weeks), and *P* = 0.0013 (24 weeks). (**C**) TAK1^ΔM^ mice developed splenomegaly. Mice (age 5–20 weeks) were euthanized, and their body weights were measured. The spleens, hearts, and kidneys were harvested and weighed; values are the mean ± SEM. Spleen, TAK1^F/+^ n = 41 and TAK1^ΔM^ n = 53; heart, TAK1^F/+^ n = 32 and TAK1^ΔM^ n = 35; Kidneys, TAK1^F/+^ n = 19 and TAK1^ΔM^ n = 27. *P<0.0001; **P<0.05. A representative image of spleens from TAK1^F/+^ and TAK1^ΔM^ mice (*inset*). (**D**) Kaplan-Meier analysis of a cohort of TAK1^F/+^ (n = 23) and TAK1^ΔM^ (n = 21) mice showing spontaneous death of TAK1^ΔM^ mice age 16–43 weeks, with a median survival duration of 29 weeks (*P*<0.0001). (**E**) Spleen weights of diseased TAK1^ΔM^ mice (n = 29) and control TAK1^F/+^ mice (n = 8).

Studies involving human patient samples were approved by the MD Anderson Institutional Review Board and the MD Anderson Ethics Committee, and in accordance with the Declaration of Helsinki, all patients gave their written informed consent to be included in the study.

**Figure 3:**
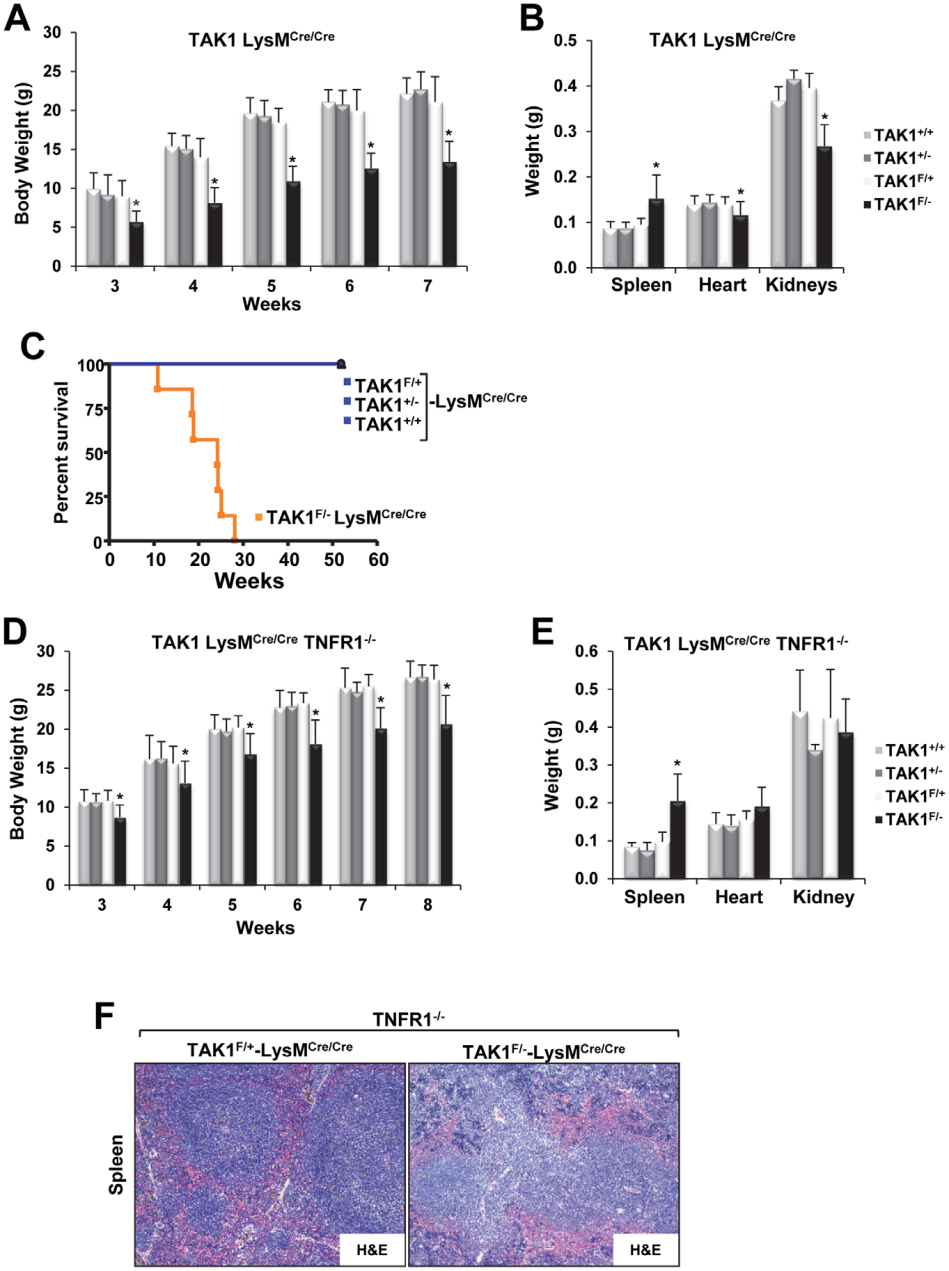
TAK1^F/−^ mice on a LysM^Cre/Cre^ background have low body weights, develop splenomegaly, and have a short life span and deletion of TNFR1 does not rescue the phenotype. (**A**) A cohort of male TAK1^+/+^ (n = 14), TAK1^+/−^ (n = 11), TAK1^F/+^ (n = 11), and TAK1^F/−^ (n = 14) mice on a LysM^Cre/Cre^ background were monitored weekly by measuring the mean (± SEM) body weight. *P<0.0001 for TAK1^F/−^ compared with all other genotypes. (**B**) A cohort of 7-week-old male TAK1^+/+^ (n = 14), TAK1^+/−^ (n = 7), TAK1^F/+^ (n = 8), and TAK1^F/−^ (n = 13) mice on a LysM^Cre/Cre^ background were euthanized, and their spleens, hearts, and kidneys were harvested and weighed; values are the mean ± SEM. *P<0.05 for TAK1^F/−^ compared with all other genotypes. (**C**) Kaplan-Meier analysis of a cohort of TAK1^F/−^ (n = 7), TAK1^F/+^ (n = 5), TAK1^+/−^ (n = 9), and TAK1^+/+^ (n = 5) mice on a LysM^Cre/Cre^ background showing spontaneous death of the TAK1^F/−^ mice 10–30 weeks of age with a median survival time of 24 weeks (*P*<0.0001). (**D**) A cohort of male TAK1^+/+^ (n = 3–5), TAK1^+/−^ (n = 4–7), TAK1^F/+^ (n = 5–8), and TAK1^F/−^ (n = 6–10) mice on a LysM^Cre/Cre^-TNFR1^−/−^ background were monitored for the indicated period, and their body weights measured. *P<0.05 for TAK1^F/−^ compared with all other genotypes. (**E**) A cohort of 6- to 8-week-old TAK1^+/+^ (n = 6), TAK1^+/−^ (n = 2), TAK1^F/+^ (n = 5), and TAK1^F/−^ (n = 9) mice on a LysM^Cre/Cre^-TNFR1^−/−^ background were euthanized, and spleens, hearts, and kidneys were harvested and weighed; values are the mean ± SEM. *P<0.05 for TAK1^F/−^ compared with all other genotypes. (**F**) Representative images of an H&E-stained spleen from TAK1^F/+^-LysM^Cre/Cre^-TNFR1^−/−^ and TAK1^F/−^LysM^Cre/Cre^-TNFR1^−/−^ littermates.

### Generation of Conditional TAK1 Knockout in the Myeloid Cell Lineage

Lysozyme M-Cre (LysMCre) knockin-mice [21] were purchased from The Jackson Laboratory (Bar Harbor, ME). Mice carrying the floxed allele of TAK1 (TAK1^F/F^) and mice carrying the null allele of TAK1 (TAK1^+/−^) have been described previously [22] and had been backcrossed to C57BL/6 mice for 6–9 generations at the time of the experiments. TAK1^F/F^-LysM^+/+^ mice were crossed with TAK1^+/−^LysM^cre/cre^ mice to generate offspring that lacked TAK1 in the myeloid lineage (TAK1^ΔM^) or were heterozygotic (TAK1^F/+^). TNFR1-null TAK1^ΔM^ mice were generated by crossing TAK1^ΔM^ mice with TNFR1-deficient mice obtained from The Jackson Laboratory. Mice were genotyped by subjecting ear clip tissues to standard polymerase chain reaction. All mice were housed in a pathogen-free animal facility. We defined young mice as those ≤8 weeks of age and old mice as those >8 weeks of age.

**Figure 4:**
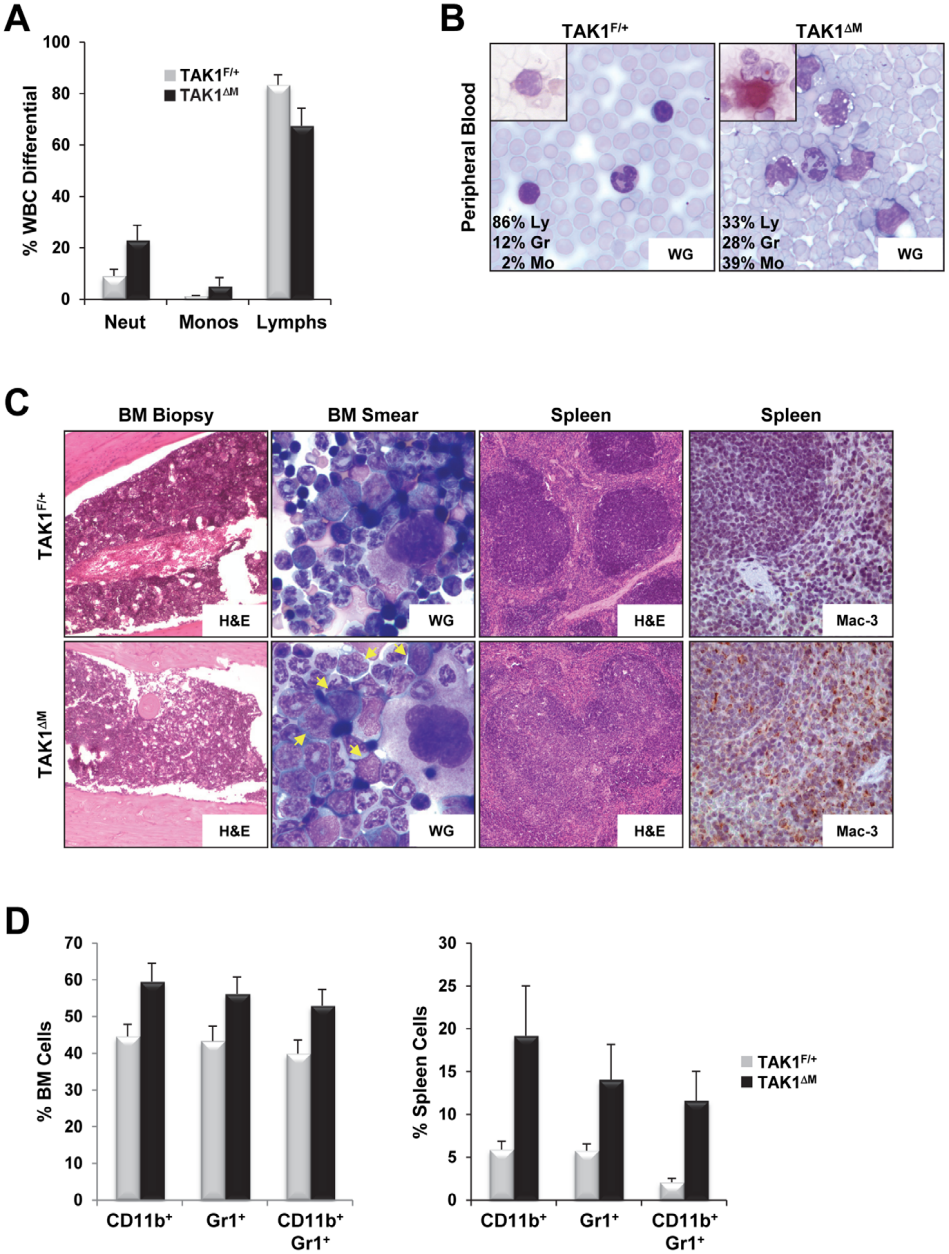
Myelomonocytic expansion in blood, bone marrow, and spleen of young TAK1^ΔM^ mice. (**A**) Increased neutrophils and monocytes in young TAK1^ΔM^ mice. Mean (± SEM) peripheral white blood cell differential counts from 6- to 8-week-old female TAK1^F/+^ (n = 7) and TAK1^ΔM^ (n = 10) mice show dramatic increases in neutrophils (*P*<0.0001) and monocytes (*P* = 0.0083) and decreases in lymphocytes (*P*<0.0001). (**B**) Peripheral blood from 7-week-old TAK1^F/+^ and TAK1^ΔM^ mice was stained with Wright-Giemsa (WG) and butyrate esterase, a brick-red monocyte-associated stain (*inset*). Also shown is the percentage of a representative manual count of lymphocytes (Ly), granulocytes (Gr), and monocytes (Mo). (**C**) Bone marrow biopsy specimens and bone marrow smears (*left panels*) from 7-week-old TAK1^F/+^ and TAK1^ΔM^ mice were stained with H&E and Wright-Giemsa, respectively. Note the increase in blasts with large folded nuclei and basophilic cytoplasm (*arrows*) in TAK1^ΔM^ mice. Spleen sections (*right panels*) from the same mice were stained with H&E and immunostained with anti-Mac-3 antibody. Note effacement of the white pulp by monotonous cell infiltrates that express Mac-3 in TAK1^ΔM^ mice. (**D**) Representative flow cytometric analysis of bone marrow (*left*) and spleen (*right*) from 7-week-old TAK1^F/+^ and TAK1^ΔM^ mice stained with Gr1 and CD11b. Graphs show the mean (± SEM) percentage of bone marrow and splenic cells (n = 5 for each genotype). For bone marrow: *P* = 0.0006, 0.0017, and 0.001 for CD11b^+^, Gr1^+^, and CD11b^+^/Gr1^+^, respectively; for spleen: *P* = 0.0011, 0.0024, and 0.0003 for CD11b^+^, Gr1^+^, and CD11b^+^/Gr1^+^, respectively.

**Figure 5:**
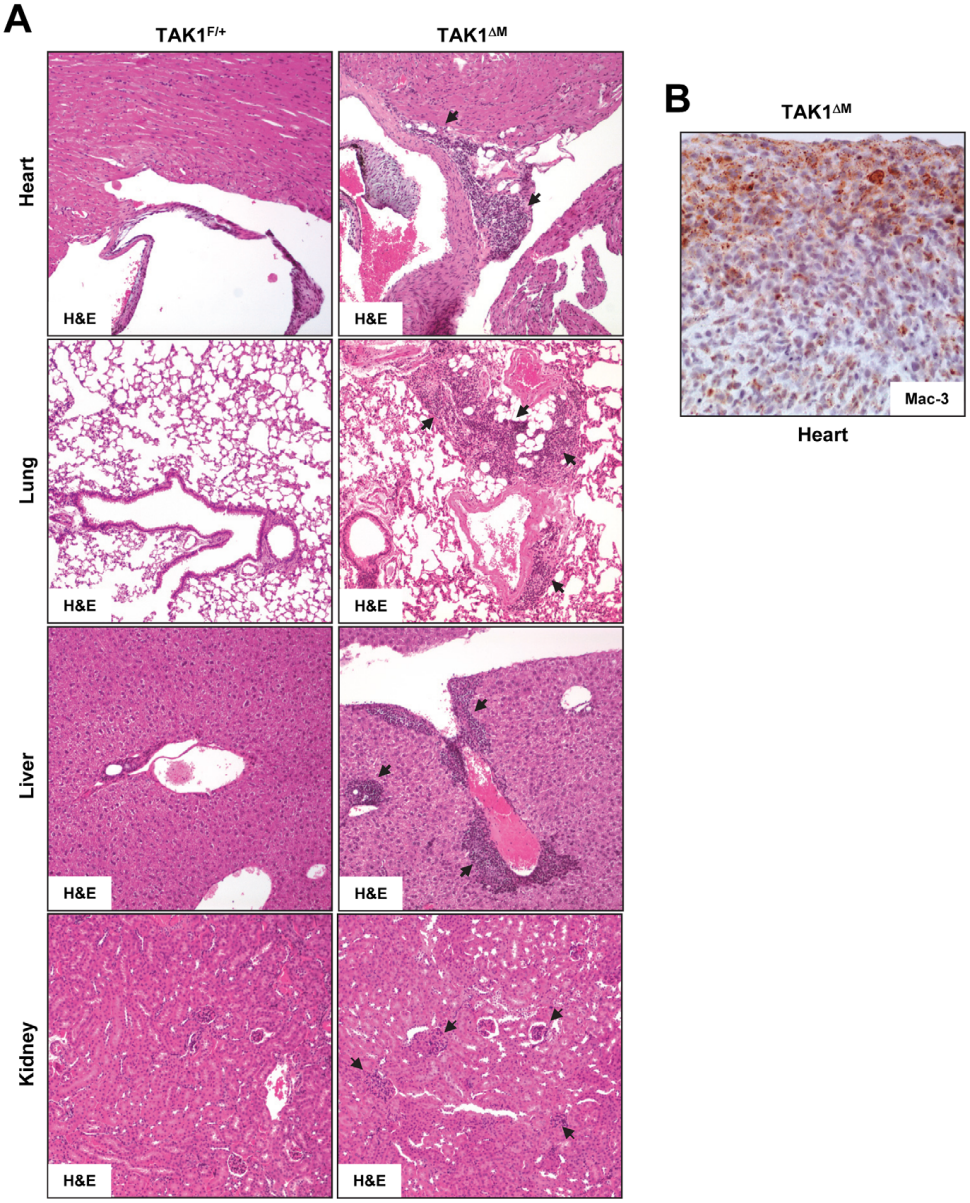
Extramedullary hematopoiesis in organs of TAK1^ΔM^ mice. (**A**) H&E-stained sections of heart, lung, liver, and kidney tissues from the indicated mice. Black arrows indicate myeloid infiltration. (**B**) The heart section from TAK1^ΔM^ mice was immunostained with an anti-Mac-3 antibody.

### Primary Mouse Monocytes Culture and Treatments

Bone marrow (BM) cells were isolated from the femurs of 6- to 10-week-old control or conditional knockout mice. The isolated cells were cultured for 24 h in α-minimal essential medium supplemented with 10% fetal bovine serum. The non-adherent cells were collected and cultured with recombinant macrophage-colony stimulating factor (M-CSF) as previously described to create bone marrow-derived monocytes (BMMs) [23], [24]. CD11b cells were isolated from BM or spleen using anti-CD11b MACS beads according to the manufacturer’s protocol (MACS Miltenyi Biotec, Cambridge, MA). Briefly, BM cells were incubated with anti-Mac1-coated microbeads and collected using MACS separation columns. To determine the signaling events induced by RANKL, LPS, M-CSF, or granulocyte macrophage stimulating factor (GM-CSF), BMMs or CD11b cells isolated from bone marrow were cultured for 4 h in medium without serum or cytokines and then stimulated with the indicated agents.

**Figure 6:**
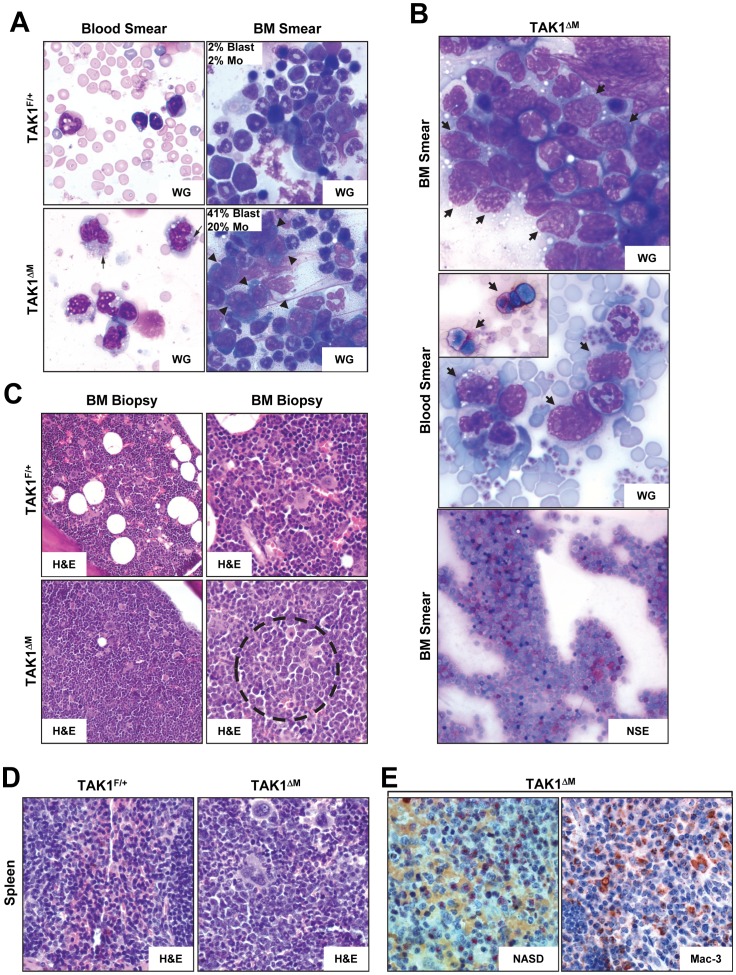
Diseased TAK1^ΔM^ mice developed myelomonocytic leukemia. (**A**) Peripheral blood smears (*left panels*) and bone marrow smears (*right panels*) from 24-week-old TAK1^F/+^ and TAK1^ΔM^ mice were stained with Wright-Giemsa (WG) and H&E, respectively. Note numerous monocytes, some with azurophilic granules (*arrows*) in the peripheral blood and increased blasts in bone marrow aspirate smears of TAK1^ΔM^ mice. Also shown for the BM smear is the percentage of a manual count of blasts and monocytes (Mo). (**B**) Blood and bone marrow smears from diseased TAK1^ΔM^ mice were stained with Wright-Giemsa (WG) and non-specific esterase (NSE). Note the increased number of blasts in the peripheral blood film and sheets of blasts in the bone marrow aspirate smear (*arrows*). A subset of blasts was positive for NSE (*inset and lower panel*). (**C**) Bone marrow biopsies from 24-week-old TAK1^F/+^ and TAK1^ΔM^ mice were stained with H&E. Note the increased number of immature cells (*dotted circle*) in the TAK1^ΔM^ mice. Magnification: left panels, 200x; right panels, 400x. (**D–E**) Tissue sections of spleens from 24-week-old TAK1^F/+^ and TAK1^ΔM^ mice were stained with H&E (**D**) and NASD (**E**, *left panel*) and immunostained with an anti-Mac-3 antibody (**E**, *right panel*).

Asynchronously growing cells were arrested in mitosis with 100 ng/ml nocodazole (Sigma, St. Louis, MO) for 12–14 hours. Mitotic cells were harvested using the mitotic shake-off method, washed in fresh medium, and released from the nocodazole block into complete medium.

**Figure 7:**
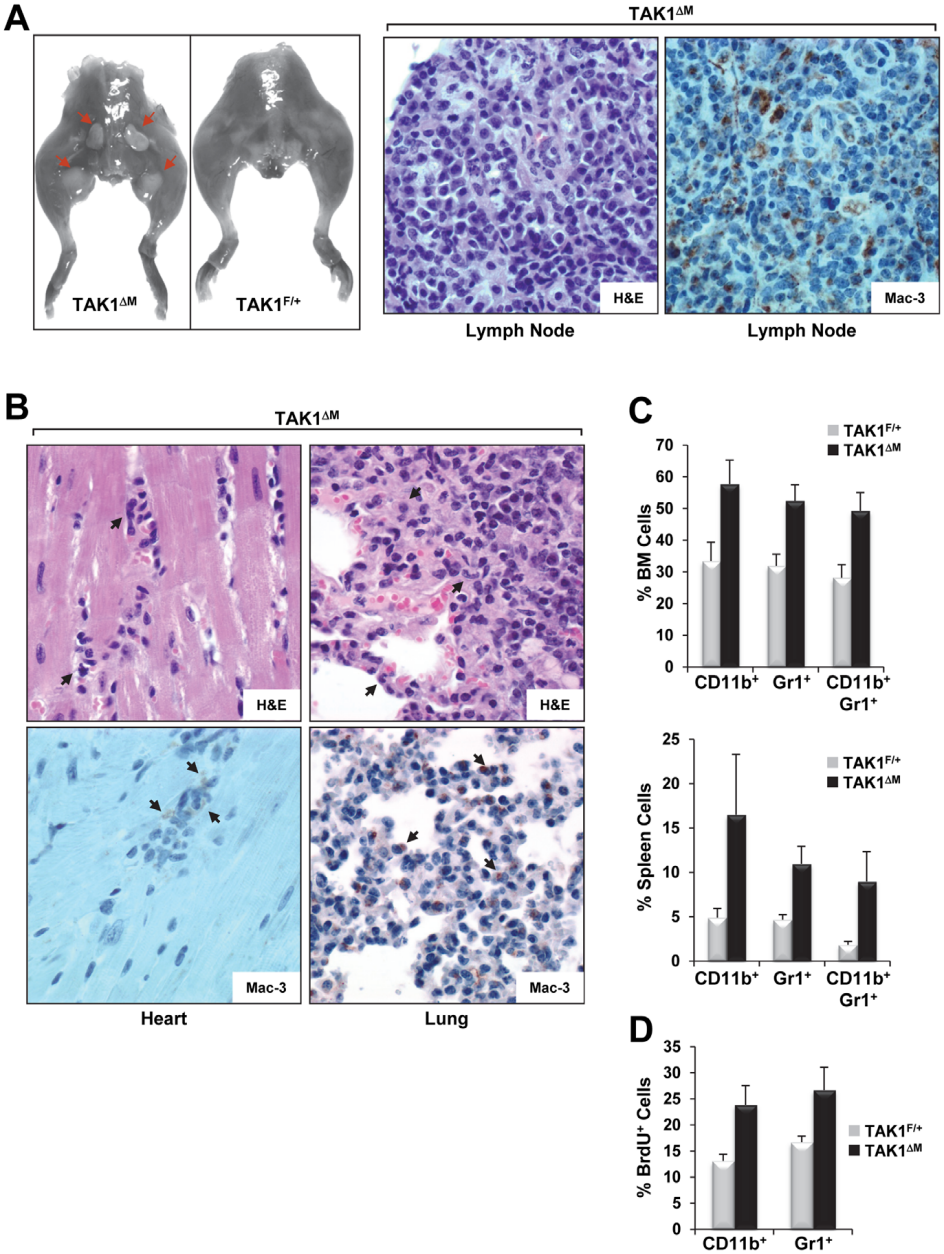
Diseased TAK1^ΔM^ mice exhibited enlarged lymph nodes and extramedullary hematopoiesis. (**A**) Photographs of the hindquarters of a 22-week-old diseased TAK1^ΔM^ mouse and a control TAK1^F/+^ littermate reveal enlarged lymph nodes (*red arrows*) in the mutant mouse (**A**, *left panel*). Tissue sections of diseased lymph nodes stained with H&E (**A**, *middle panel*) and immunostained with an anti-Mac-3 antibody (**A**, *right panel*). (**B**) Heart and lung tissue sections of a diseased TAK1^ΔM^ mouse stained with H&E and immunostained with an anti-Mac-3 antibody, as indicated. (**C**) Flow cytometric analysis of bone marrow (*top*) and splenocytes (*bottom*) from 24-week-old TAK1^F/+^ and TAK1^ΔM^ mice stained with Gr1 and CD11b. Graphs show the mean (± SEM) percentage of bone marrow and splenic cells (n = 4 for each genotype). For bone marrow: *P* = 0.0023, 0.0006, and 0.0010 for CD11b^+^, Gr1^+^, and CD11b^+^/Gr1^+^, respectively; for spleen: *P* = 0.0150, 0.0008, and 0.0054 for CD11b^+^, Gr1^+^, and CD11b^+^/Gr1^+^, respectively. (**D**) Flow cytometric analysis of bone marrow from the indicated mice pulse labeled with BrdU and gated for incorporation of BrdU in the CD11b and Gr1 cell populations (*P*<0.02).

### Cell Lines, Retroviral Vectors, Antibodies, and Reagents

The retroviral packaging cell line GP2-293 was purchased from Clontech (Mountain View, CA) and cultured as described previously [23]. TAK1-knockout (KO) mouse embryonic fibroblasts (MEFs) were obtained by transiently expressing Cre recombinase in immortalized TAK1^F/F^ MEFs using the 3T3 method and validated for loss of TAK1 via immunoblotting and genomic polymerase chain reaction. TAK1-KO MEFs expressing exogenous FLAG-tagged TAK1-WT or TAK1-K63A were generated using retroviral infection with a pMX-IRES-GFP vector containing a puromycin-selectable marker [23], [25]. Recombinant RANKL, GST-cJun and 6x-His-tagged MKK6 kinase-dead have been described previously [23], [24], [26], and recombinant GM-CSF was purchased from PeproTech (Rocky Hill, NJ). Antibodies against TAK1, JNK, cyclin B1, p65, p50, TAB2, cIAP1, Bcl-xL, Bcl-2, phospho-Stat3, Stat3, and ubiquitin were purchased from Santa Cruz Biotechnology (Santa Cruz, CA); IKKα, IKKβ, and IKKγ were from Imgenex (San Diego, CA); TRAF6 and phospho-tyrosine were from Millipore (Billerica, MA); phospho-p38, phospho-IκBα, phospho-Erk1/2, Erk1/2, phospho-Stat5, Stat5, p(Ser10)-histone H3, phospho-AKT, and AKT were from Cell Signaling Technology (Danvers, MA); FLAG was from Sigma (St. Louis, MO); Aurora A and goat anti-mouse immunoglobulin G-conjugated horseradish peroxidase were from BD Biosciences (San Jose, CA); goat anti-rabbit immunoglobulin G-conjugated horseradish peroxidase was from Bio-Rad (Hercules, CA); and β-actin was from Cytoskeleton (Denver, CO).

**Figure 8:**
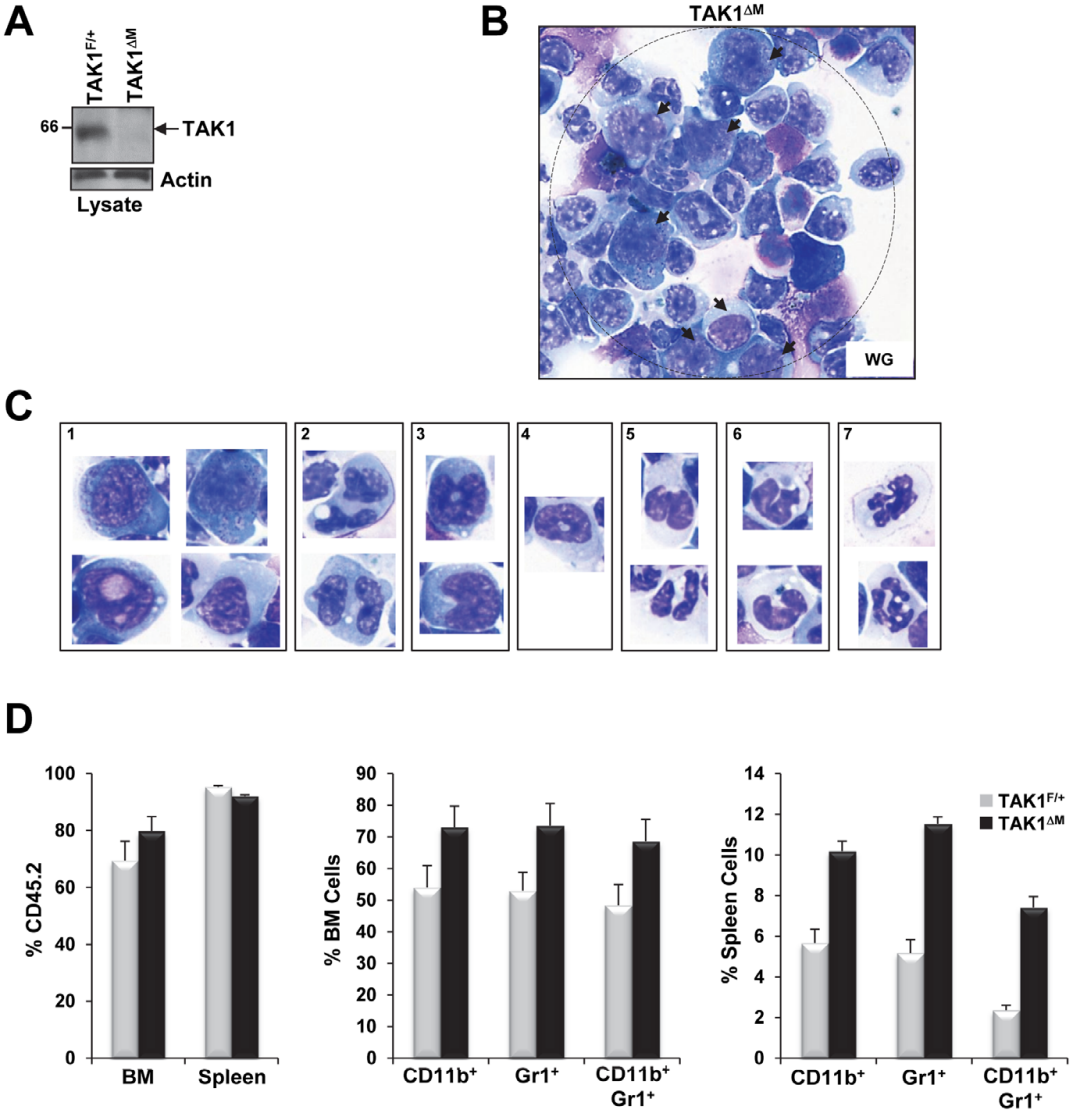
Increase blasts in CD11b^+^ splenocytes of diseased TAK1^ΔM^ mice and expansion of the myelomonocytic compartment in recipient mice transplanted with TAK1^ΔM^ bone marrow. (**A**) Western blot analysis revealed absence of TAK1 protein in CD11b^+^ splenic cells of diseased TAK1^ΔM^ mice. Splenocytes from the indicated mice were harvested, and CD11b^+^ cells were sorted by magnetic beads. Cell lysates were subjected to SDS-PAGE and immunoblotted with the indicated antibodies. (**B–C**) CD11b^+^ splenocytes of diseased TAK1^ΔM^ mice revealed high occurrence of blasts as well as monocytes and granulocytes with abnormal features. Cytospins of CD11b^+^ splenocytes of diseased TAK1^ΔM^ mice were stained with Wright-Giemsa. Note the presence of blasts (**B,** *arrows*; **C**, *panel 1*), abnormal multinucleated myeloid forms (**C**, *panel 2*), abnormal monocytes (**C**, *panel 3*), dysplastic hypogranular granulocytes (**C**, *panel 4*), hypogranular Pelger neutrophils (**C**, *panel 5*), dysplastic neutrophils (**C**, *panel 6*), hypergranulated neutrophils (**C**, *panel 7*). (**D**) Increase in monocytic and granulocytic cells after transplant of TAK1^ΔM^ bone marrow into recipient mice. Flow cytometric analysis of bone marrow (*middle graph*) and spleen (*right graph*) from 28-week-old TAK1^F/+^ or TAK1^ΔM^ mice that underwent bone marrow transplantation and were stained for Gr1 and CD11b from cells gated for expression of CD45.2. Graphs show the mean (± SEM) percentage of CD45.2^+^ cells in bone marrow and spleen (*left*) or bone marrow (*middle*) and splenic cells (*right*) (n = 3 for each genotype). For bone marrow: *P* = 0.0263, 0.0174, and 0.0209 for CD11b^+^, Gr1^+^, and CD11b^+^/Gr1^+^, respectively. For spleen: *P* = 0.0007, 0.0001, and 0.0001 for CD11b^+^, Gr1^+^, and CD11b^+^/Gr1^+^, respectively.

### Immunoblotting, Immunoprecipitation, and Kinase Assays

Cell lysis, immunoblotting, immunoprecipitation, and *in vitro* kinase assays were performed as described previously [23], [24], [26].

**Figure 9:**
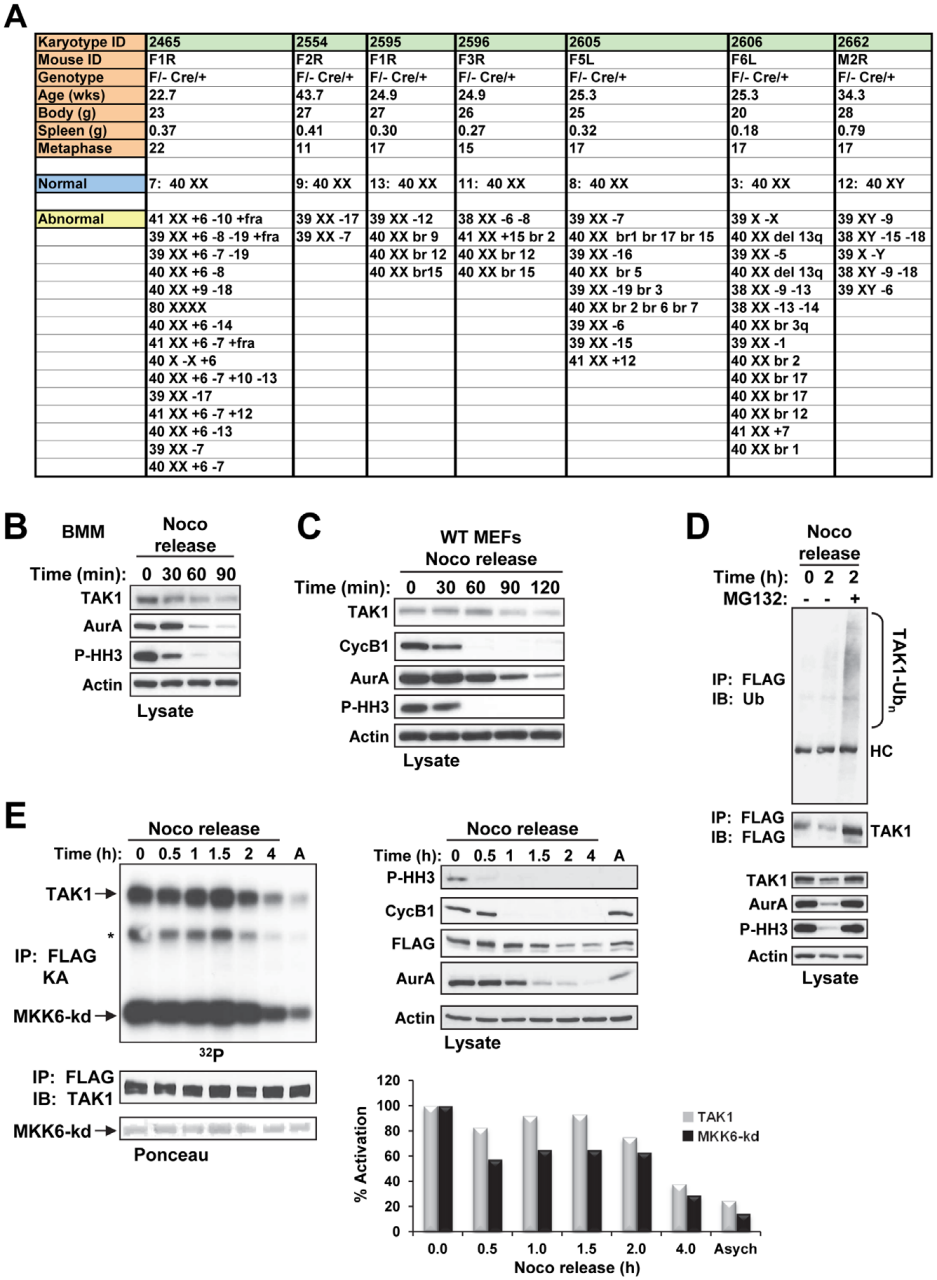
Diseased TAK1^ΔM^ Mice Exhibit Genomic Instability and TAK1 is regulated during mitosis. (**A**) TAK1^ΔM^ mice were euthanized because of morbidity, and body and spleen weights were measured. Bone marrow was harvested, and short-term cultures were collected and processed for G-banding using standard methods. Analysis of 11–22 metaphase cells per mouse was performed, and the results listed indicated chromosomal deletions, additions, or breaks. (**B–C**) Primary mouse BMMs (**B**) or TAK1-WT MEFs (**C**) were treated with nocodazole (Noco, 100 ng/ml) for 14 h. Mitotic cells were collected by the mitotic shake-off method and released into fresh medium for the indicated times. Cell lysates were subjected to SDS-PAGE and immunoblotted with the indicated antibodies. (**D**) TAK1-KO MEFs stably expressing FLAG-TAK1 were treated with nocodazole (100 ng/ml) for 14 h. Mitotic cells were collected by the mitotic shake-off method and released into fresh medium for the indicated times in the absence or presence of MG132 (25 µM; added 30 min after nocodazole release). Cell lysates were subjected to SDS-PAGE and immunoblotted with the indicated antibodies (*bottom panels*). Protein lysates were immunoprecipitated with anti-FLAG and immunoblotted with anti-ubiquitin (Ub) as indicated (*top panel*). The membrane was stripped and reprobed with anti-FLAG (*middle panel*). (**E**) TAK1-KO MEFs stably expressing FLAG-TAK1 were left untreated (A; asynchronized) or treated with nocodazole (100 ng/ml) for 14 h. Mitotic cells were collected by the mitotic shake-off method and released into fresh medium for the indicated times. Cells lysates were subjected to SDS-PAGE and immunoblotted with the indicated antibodies (*right panels*). Protein lysates were immunoprecipitated with anti-FLAG and an *in vitro* kinase assay with kinase-dead MKK6 (MKK6-kd) as the substrate was performed. The amount of total protein was adjusted so that an equivalent amount of TAK1 was immunoprecipitated from each time. The samples were subjected to SDS-PAGE, transferred to a membrane, and exposed to X-ray film (*upper left panel*). The membrane was stained with Ponceau S (*bottom left panel*) and probed with anti-TAK1 (*middle left panel*). Incorporation of ^32^P into the substrate and TAK1 was quantitated using a PhosphoImager and presented as percent activation (*graph*). Asych, asynchronized.

### Histopathologic, Immunohistochemical, Cytochemical, and Hematopathologic Analyses

Organs were removed from TAK1^F/+^ and TAK1^ΔM^ mice, dissected, and fixed in 10% neutral-buffered formalin (Sigma). Femurs removed from these mice were decalcified and embedded in paraffin. Deparaffinized tissue sections were stained with hematoxylin and eosin (H&E). An immunohistochemical analysis was performed using an anti-Mac-3 antibody (BD Biosciences) according to standard procedures. Cytochemical staining for Wright-Giemsa, myeloperoxidase, and butyrate esterase and chloroacetate esterase (all from Sigma) was performed according to the manufacturer’s protocols. Peripheral blood was collected by heart puncture immediately after the mice were killed, and complete blood cell counts were determined using an automated Hemavet hematologic analyzer in the MD Anderson’s Department of Veterinary Medicine and Surgery Histopathology Core.

**Figure 10:**
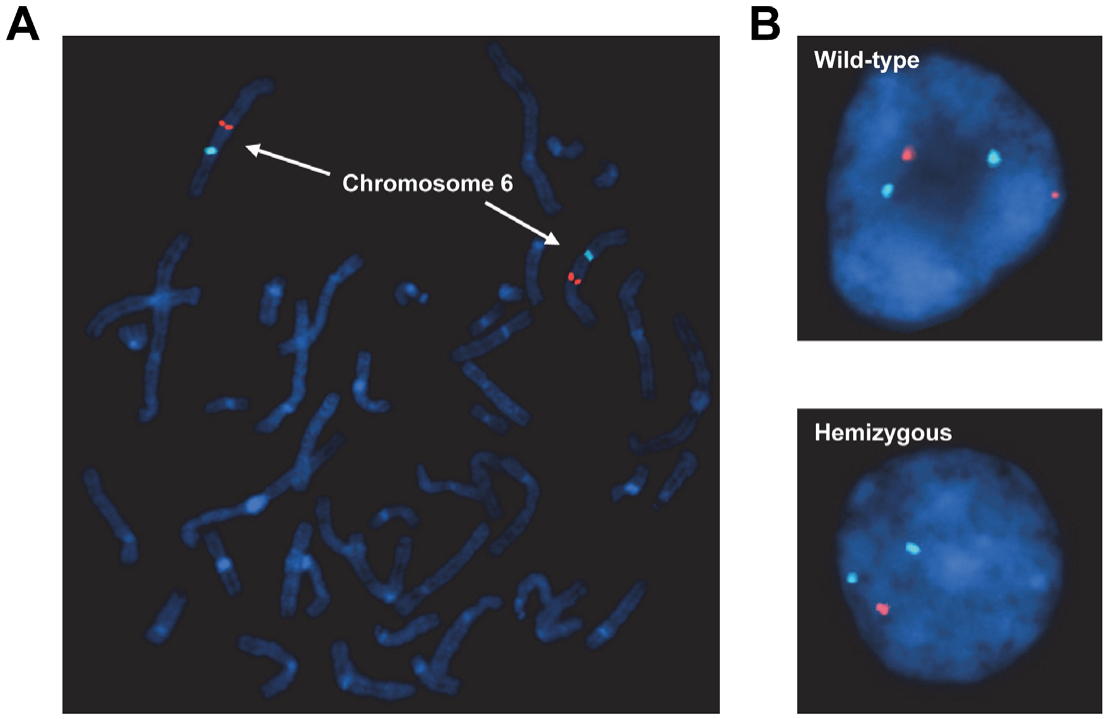
Detection of human TAK1 gene on chromosome 6 by FISH. (**A**) A metaphase spread shows CEP6 (aqua) and TAK1 (orange) highlighted on chromosome 6. (**B**) Examples of interphase cells probed with CEP6 and TAK1 that were scored as TAK1-WT (*top panel*) and TAK1-hemizygous (*bottom panel*).

**Table 1:** TAK1 deletion and clinical features of leukemia patients.

Patient	Age/Sex^1^	Blast %	Diagnosis^2^	Genetic Group^3^	TAK1 FISH^4^
1	59/M	40	AML	Favorable	Positive
2	53/F	20	AML	Favorable	Positive
3	33/F	24	AML	Favorable	Negative
4	69/M	84	AML	Intermediate-I	Negative
5	70/F	54	AML	Intermediate-I	Negative
6	73/F	41	AML	Intermediate-I	Positive
7	71/F	54	AML	Intermediate-I	Negative
8	75/M	70	AML	Intermediate-I	Positive
9	80/M	26	AML	Not available	Positive
10	60/M	36	AML	Intermediate-II	Negative
11	69/M	26	AML	Intermediate-II	Positive
12	26/M	93	AML	Intermediate-II	Positive
13	75/F	23	AML	Intermediate-II	Negative
14	70/M	91	AML	Intermediate-II	Positive
15	81/F	30	AML	Intermediate-II	Negative
16	79/M	31	AML	Intermediate-II	Negative
17	66/M	36	AML	Intermediate-II	Negative
18	70/M	82	AML	Intermediate-II	Negative
19	26/M	26	AML	Adverse	Positive
20	40/M	21	AML	Adverse	Positive
21	62/F	23	AML	Adverse	Positive
22	72/M	22	AML	Adverse	Negative
23	64/M	82	AML	Adverse	Negative
24	51/F	40	AML	Adverse	Negative
25	55/M	77	AML	Adverse	Negative
26	60/F	24	AML	Adverse	Positive
27	77/M	23	AML	Adverse	Negative
28	28/M	50	AML	Adverse	Positive
29	64/F	65	AML	Adverse	Negative
30	47/M	25	AML	Adverse	Negative

1 M, male; F, female.

2 AML, acute myeloid leukemia.

3 Genetic group is designated as described [52].

4 Positive reflects greater than 6% (cutoff value) abnormal cells from 200 interphase cells scored by FISH analysis as having no TAK1 allele (2A0R) or only one TAK1 allele (2A1R).

### Flow Cytometric Analysis

Bone marrow cells were flushed from the femurs and tibias of mice, and splenic or other cells were dissociated into single cells with collagenase. Red blood cells were lysed using hypotonic buffer, and single-cell suspensions were incubated with Fc-block and stained with a mixture of fluorescence-conjugated antibodies. Antibodies against CD11b (M1/70), Gr1 (RB6-8C5), CD43 (e-BioR2/60), F480 (BM8), B220 (RA3-6B2), IgM (R6-60.2), CD4 (GK1.5), CD8 (53-6.7), CD45.1, and CD45.2 were purchased from e-Bioscience (San Diego, CA) or BD Biosciences (San Jose, CA). Flow cytometric data were collected using fluorescence-activated cell sorting with a FACSCanto or LSRII (BD Biosciences) and analyzed using the FlowJo software program (version 10.0 Tree Star, Inc., Ashland, OR).

### In vivo Proliferation Assay

Mice were injected intraperitoneally with 100 mg/kg BrdU and killed 6 h after injection. BM cells and splenocytes were stained with APC-CD11b and PercP-Gr1, fixed, and stained using the FITC BrdU Flow Cytometry kit (BD Bioscience) before being analyzed by flow cytometry using the FlowJo software program.

### Generation of Radiation Chimeras

BM cells (2 × 10^6^ per recipient) harvested from the femurs of TAK1^ΔM^ or TAK1^F/+^ mice (Ly5.2), were injected into the tail veins of C57BL/6-Ly5.1 mice (The Jackson Laboratory) that had been exposed to a lethal dose of radiation (10 Gy). Peripheral blood samples were regularly harvested to monitor the mice’s white blood cell counts. The mice were killed 28 weeks after the injection of BM cells, and hematologic, histologic, and flow cytometric analyses of these mice were performed.

### Cytogenetic Analysis

A standard conventional cytogenetic analysis was performed on diseased TAK1^ΔM^ bone marrow samples that were cultured in the presence of GM-CSF and IL-3 for a short period and then subjecting the samples to G-band karyotyping. Cytogenetic analysis was performed at MD Anderson’s Molecular Cytogenetics core facility.

### TAK1/MAP3K7 Detection by Fluorescence in situ Hybridization Analysis

Two bone marrow samples were obtained from each of 39 patients diagnosed with AML and CMML at The University of Texas MD Anderson Cancer Center. Deletion of the *MAP3K7* gene was analyzed by fluorescence *in situ* hybridization **(**FISH) analysis with the PAC clone RP1-154G14, which contains the entire TAK1 gene (BACPAC, http://bacpac.chori.org). The RP1-154G14 clone was labeled using nick translation with dUTP SpectrumOrange (Abbott Molecular, Des Plaines, IL) and chromosome 6 was enumerated using a commercial centromeric probe, CEP6, labeled with SpectrumAqua (Abbott Molecular). Five normal metaphase slides were analyzed to validate the RP1-154G14 and CEP6 probes. The RP1-154G14 probe was validated by using CytoVision software program (version 4.5.2, Buffalo Grove, IL) to map the probe back to chromosome 6q15 on five normal control metaphase slides using standard FISH procedures. Fluorescent images of DAPI-inverted and non-DAPI-inverted metaphase cells were obtained for each slide.

To establish the normal cut-off level of TAK1 deletion, we fixed five normal peripheral blood smear slides and evaluated them using FISH analysis. Two separate individuals counted 100 interphase cells with the appropriate SpectrumOrange and SpectrumAqua filters; these stained cells were scored as normal 2A2R (A, aqua; R, orange), single allelic deleted 2A1R, or bi-allelic deleted 2A0R. The normal cut-off level was established using the β inverse function of the Microsoft Excel software program (version 2010, Microsoft, Redmond, WA), as described previously [27]. The result was a 5.05% cut-off, or 10.1 cells. Because fractions of cells could not be analyzed, the cut-off level was rounded to 11, cells or 5.5% of 200 cells. At the 95% confidence level, the abnormal cut-off level was 11 cells (*i.e.*, 12 or more cells of 200 cells analyzed would be considered an abnormal result). Once this had been established, the remaining specimens were analyzed. For *MAP3K7* deletion analysis, 100 interphase cells were counted by two separate counters independently for each patient using the same scoring system for the normal cut-off level. Because each patient’s slide was evaluated twice, the final percentage of abnormal cells was averaged between the two slides [27].

### Data Analysis

All values are expressed as means ± standard errors of the mean (SEM) unless otherwise indicated. Differences between experimental groups were assessed using an unpaired two-tailed Student *t*-test (GraphPad). *P* values <0.05 were considered statistically significant.

## Results

### TAK1 Deletion in the Myeloid Lineage Impairs Cytokine-mediated Signaling

To examine the function of TAK1 in hematopoietic cells of the myeloid lineage, we used mice carrying a floxed TAK1 allele [22], in which the first exon of TAK1 containing the start codon is flanked by loxP sites (TAK1^F/F^), and lysozyme M-Cre (LysM-Cre) mice that express Cre recombinase under the control of the lysozyme M promoter (LysM^Cre/Cre^) [21]. The LysM-Cre line expresses Cre exclusively in hematopoietic cells of the myelomonocytic lineage, with expression being at a moderate level in committed myeloid progenitors and at a high level in mature monocytes, macrophages, and neutrophils [21]. TAK1^F/F^ LysM^+/+^ mice were crossed with TAK1^+/−^ LysM^Cre/Cre^ to produce littermates that were TAK1^F/+^ LysM^Cre/+^ (referred to as TAK1^F/+^) and TAK1^F/−^ LysM^Cre/+^ (referred to as TAK1^ΔM^, deleted in myeloid lineage) (**Fig. 1A**). We found no significant gross morphological differences between heterozygote (TAK1^+/−^ LysM^Cre/+^; TAK1^F/+^ LysM^Cre/+^) and wild type (TAK1^+/+^ LysM^Cre/+^) mice, which is consistent with previous reports [12], [14], [15], [17]. Western blot analysis confirmed the deletion of TAK1 in CD11b^+^ cells from the bone marrow of TAK1^ΔM^ mice (**Fig. 1B**).

Previous reports have indicated that TAK1 is required for IKK and stress kinase activation induced by various stimuli including IL-1β, TNFα, LPS, and RANKL [15], [25], [28]. To determine whether TAK1 deletion prevents stimulus-dependent IKK and JNK activation, we generated M-CSF-dependent bone marrow-derived monocytes (BMMs) from TAK1^F/+^ and TAK1^ΔM^ littermates. Following stimulation with RANKL, BMMs from TAK1^ΔM^ mice had impaired activation of both IKK and JNK, as revealed by the level of IκBα phosphorylation and an *in vitro* kinase assay, respectively (**Fig. 1C**). Similarly, following stimulation with LPS, BMMs from TAK1^ΔM^ mice had diminished activation of IKK and JNK (**Fig. 1D**). In contrast, M-CSF treatment of BMMs and GM-CSF treatment of sorted CD11b^+^ cells did not reveal any reproducible differences between cells from TAK1^F/+^ and TAK1^ΔM^ mice, indicating that loss of TAK1 does not affect M-CSF or GM-CSF signaling (**Fig. 1E–F**). Furthermore, monocytes from the TAK1^F/+^ and TAK1^ΔM^ mice had similar expression levels of a number of signaling proteins in the TAK1 signaling pathway (**Fig. 1G**). Together, these results confirm that TAK1 is deleted in the myeloid lineage, TAK1-deficient monocytes fail to respond to stimulation with RANKL or LPS, and TAK1 is not involved in the M-CSF or GM-CSF signaling pathways.

### TAK1 Deletion in the Myeloid Lineage Results in Growth Retardation, Splenomegaly, and Shortened Life Span

Regardless of sex, TAK1^ΔM^ mice had lower body weights than their control littermates (**Fig. 2A–B**). A comparative analysis of cohorts of TAK1^F/+^ and TAK1^ΔM^ mice at various ages revealed that TAK1^ΔM^ mice had severe splenomegaly and the weights of the hearts and kidneys from TAK1^F/+^ and TAK1^ΔM^ mice did not differ significantly (**Fig. 2C**). Unexpectedly, older TAK1^ΔM^ gained less weight and exhibited more lethargy compared with their TAK1^F/+^ littermates; the median survival duration of TAK1^ΔM^ mice was 29 weeks (range, 16–43 weeks) (**Fig. 2D**). In contrast, the TAK1^F/+^ mice thrived for 14-months. The weight of the spleens of the diseased TAK1^ΔM^ mice that developed severe morbidity was significantly higher than that of control littermates (**Fig. 2E**).

Because the TAK1^ΔM^ mice had a severe and fatal phenotype, we decided to compare all TAK1 genotypes by breeding TAK1-floxed mice with LysM-Cre mice with both copies of the *Cre* allele (*i.e.*, LysM^Cre/Cre^), in which the breeding scheme facilitated the comparison of all TAK1 genotypes (*i.e.*, TAK1^F/−^, TAK1^F/+^, TAK1^+/−^, and TAK1^+/+^). The attributes of the TAK1^F/−^LysM^Cre/Cre^ mice – reduced body weight, splenomegaly, and shorter life span – were similar to those of the TAK1^ΔM^ mice, and the TAK1^F/−^LysM^Cre/Cre^ mice had a median survival time of 24 weeks (**Fig. 3A–C**). The phenotypes of the other TAK1-LysM^Cre/Cre^ genotypes (*i.e.*, TAK1^F/+^, TAK1^+/−^, and TAK1^+/+^) were similar, demonstrating that the phenotype of the TAK1^ΔM^ mice was not due to the presence of Cre (**Fig. 3A–C**). Furthermore, previous studies have demonstrated that simultaneous deletion of TNFR1 can significantly attenuate the severe pathologies observed in TAK1 conditional knockout mice [4], [6], [7]. Therefore, we sought to determine whether TNFR1 deletion could rescue the phenotype in TAK1^F/−^LysM^Cre/Cre^ mice. The simultaneous deletion of TNFR1 did not modify the severe phenotype that developed in TAK1^F/−^LysM^Cre/Cre^ mice; in fact, TAK1^F/−^LysM^Cre/Cre^ mice on a TNFR1-null background developed splenomegaly and died at 25–43 weeks of age (**Fig. 3D–F**). Collectively, these results indicate that loss of TAK1 in the myeloid lineage results in a 100% penetrable fatal disease.

### Expansion of the Myelomonocytic Compartment in Young TAK1^ΔM^ Mice

We next evaluated the myeloid compartment in peripheral blood, bone marrow, spleen, and other organs in 7- to 8-week-old mice. Peripheral white blood cell counts revealed dramatic increase in neutrophils and monocytes and a moderate decrease in lymphocytes in young TAK1^ΔM^ mice (**Fig. 4A**). The absolute neutrophil count in TAK1^ΔM^ mice (2505±1034 cells/µl) was significantly higher than of their TAK1^F/+^ littermates (613±199 cells/µl; *P*<0.0002). Peripheral blood smears revealed that the occurrence of esterase-positive monocytes in TAK1^ΔM^ mice was significantly higher than in the TAK1^F/+^ littermates (**Fig. 4B**). H&E and Wright-Giemsa staining revealed left-shifted granulopoiesis, with increased numbers of immature myeloid and blast cells in the bone marrow from TAK1^ΔM^ mice (**Fig. 4C, *left panel*** ***s***). Histological analysis revealed disruption of the splenic architecture in TAK1^ΔM^ mice, as evidenced by large areas of mature and immature myeloid-appearing cells in the red pulp and a marked increase in the white pulp of cells that resembled maturing monocytes that was confirmed by positive staining for Mac-3 (**Fig. 4C, *right panel*** ***s***). Fluorescence-activated cell sorting (FACS) confirmed the increase in Gr1, CD11b, and Gr1/CD11b double-positive cells in the bone marrow and spleen of young TAK1^ΔM^ mice (**Fig. 4D**). H&E stained tissue sections showed massive infiltration of myeloid cells specifically in the lungs, heart, liver, and kidneys, indicating prominent extramedullary hematopoiesis in TAK1^ΔM^ mice (**Fig. 5A**). Positive staining for Mac-3 confirmed these cells’ monocytic origin (**Fig. 5B**).

### Leukemic Disease Progression in Aged TAK1^ΔM^ Mice

Wright-Giemsa staining of peripheral blood smears confirmed the presence of increased numbers of granulocytic and monocytic cells, some with immature nuclei and distinct nucleoli in older diseased TAK1^ΔM^ mice (**Fig. 6A, *left panel*** ***s***). Wright-Giemsa staining of bone marrow aspirate smears revealed foci of blast cells with large nuclei, distinct nucleoli, and basophilic cytoplasm in these mice (**Fig. 6A, *right panel*** ***s,*** **6B,** ***top panel***). The immature forms or blasts in the peripheral blood and bone marrow smears stained positive for butyrate esterase, confirming these cells’ monocytic lineage (**Fig. 6B** **,** ***middle and bottom panel*** **s**). Bone marrow biopsy samples from TAK1^F/+^ mice were 80% cellular with a tri-lineage population of mature and maturing cells, whereas bone marrow biopsy samples from TAK1^ΔM^ mice were 100% cellular with a monotonous population of immature myeloid cells (**Fig. 6C**). Histopathologic analyses of spleen samples from TAK1^ΔM^ mice revealed the complete destruction of the organ architecture by monotonous cells, which were large with irregular nuclear contours and stained positive for NASD and Mac-3, confirming the myelomonocytic lineage of the leukemic blasts (**Fig. 6D–E**).

Unlike TAK1^F/+^ mice, leukemic TAK1^ΔM^ mice had multiple enlarged lymph nodes. In TAK1^ΔM^ mice, H&E staining revealed lymph node architecture effacement by an extensive leukemic cell infiltrate composed of various maturing and immature monocytoid-looking forms with large folded nuclei that were undergoing apoptosis and mitosis and stained positive for Mac-3 (**Fig. 7A**). An examination of other organs, including the heart, lungs, liver, and kidneys, also revealed leukemic blasts that stained positive for Mac-3 (**Fig. 7B**). Flow cytometric analysis of bone marrow and splenic cells revealed expansion of Gr1, CD11b, and Gr1/CD11b double-positive cells (**Fig. 7C**), which was consistent with histopathologic examinations that revealed the leukemic blasts’ increased granulocytic and monocytic nature. In addition, BrdU-labeling revealed that Gr1 and CD11b cells from bone marrow had increased proliferation capacity *in vivo* (**Fig. 7D**). Wright-Giemsa staining of CD11b^+^ splenocytes of diseased TAK1^ΔM^ mice that showed decreased TAK1 expression (**Fig. 8A**) revealed a high occurrence of leukemic blast cells as well as monocytes and neutrophils with abnormal features (**Fig. 8B–C**). In contrast, CD11b^+^ splenocytes of older TAK1^F/+^ mice revealed normal mature monocytes and granulocytes (data not shown).

Whole bone marrow transplanted from TAK1^ΔM^ mice into lethally irradiated mice resulted in a similar myelomonocytic neoplasm. Of the 7 mice that received bone marrow from TAK1^ΔM^ mice, 4 died 11, 21, 25, and 26 weeks, respectively, after transplantation. The remaining 3 TAK1^ΔM^ recipient mice had increased Gr1, CD11b, and Gr1/CD11b double-positive cells 28 weeks after transplantation, as assessed by gating on CD45.2-positive cells and compared with mice receiving BM from TAK1^F/+^ mice (**Fig. 8D**). These results suggest that the phenotype of myeloid-specific TAK1 ablation is cell-autonomous. On the basis of the criteria for classifying nonlymphoid hematopoietic neoplasms in mice [29], our findings demonstrate that TAK1^ΔM^ mice developed fatal myeloid leukemia with myelomonocytic characteristics and features that are similar to those of human myelomonocytic leukemia.

### Genomic Instability in TAK1^ΔM^ Mice and Regulation of TAK1 Expression and Activity during Mitosis

Because genomic instability is an integral component of myeloid leukemia [30], we sought to determine whether leukemic cells from TAK1^ΔM^ mice had acquired chromosomal abnormalities. We found that each of the TAK1^ΔM^ mice had chromosomal abnormalities, including deletions, additions, and chromosomal breaks (**Fig. 9A**). These observations are consistent with those of Betterman *et al.*, who found that liver tumors that arise from the conditional ablation of TAK1 in hepatocytes lead to chromosomal aberrations, suggesting that loss of TAK1 is linked to genomic instability [8]. However, the authors did not investigate the relationship between TAK1 loss and chromosome integrity.

Recently, a siRNA kinome screen identified TAK1 among 779 kinases as one of the top mitotic progression regulators whose loss increased the ploidy index at a similar level to known mitotic kinase regulators such as BUB1, BUB1B, CDC2, PLK1, PLK3, and TTK (MPS1) [31]. On the basis of these observations, we sought to determine whether TAK1 is regulated during the mitotic phase of the cell cycle. Treatment with nocodazole synchronizes cells in early mitosis as rounded, floating cells, which can be isolated and released into fresh medium to complete the cell cycle; the disappearance of Aurora A, cyclin B, and phospho-histone H3 on Ser10 serve as mitotic markers. In nocodazole-released BMM or MEFs, TAK1 expression, like that of the mitotic markers, decreased over time (**Fig. 9B–C**), which suggests that TAK1 is ubiquitinated and degraded as the cells progress through mitosis. Indeed, TAK1 was protected from degradation in the presence of the proteasomal inhibitor MG132 (**Fig. 9D** **,** ***bottom panels***) and was polyubiquitinated (**Fig. 9D** **,** ***top panel***).

We next determined whether changes occur in the kinase activity of TAK1 during mitosis. Immunoprecipitation of FLAG-TAK1 from reconstituted TAK1 KO MEFs followed by an *in vitro* kinase assay using kinase-dead MKK6 as a substrate revealed that nocodazole-arrested cells had the highest TAK1 kinase activity (5-fold higher than that of asynchronized cells) that, when the cells were released into fresh medium, began to decrease over time and approached the level of activity similar to that in asynchronized cells (**Fig. 9E** **,** ***top panel and graph***). Catalytically inactive TAK1 (K63A mutant) failed to phosphorylate MKK6-KD under the same conditions (**data not shown**). Collectively, these data reveal that TAK1 protein stability and activity are regulated during the mitotic phase of the cell cycle.

### TAK1 Gene Deletion is Prevalent in AML Patients

As the TAK1^ΔM^ mice aged, they developed a CMML-like disease that transformed to AML, which is reminiscent of the clinical progression of CMML in humans [32]. Therefore, we sought to determine whether the human TAK1 gene located at 6q15–16 is deleted in a subset of patients with myeloid leukemia. We examined bone marrow aspirate samples from 30 patients with AML using two-color FISH analysis with a probe that contains the entire TAK1 genomic locus and a centromeric probe to chromosome 6. An evaluation of a metaphase spread revealed positive staining for chromosome 6 confirming the specificity of the TAK1 probe (**Fig. 10**). FISH analysis revealed prevalent deletion of the TAK1 gene in 13 of 30 (43%) AML patients (**Table 1**) and 2 of 9 (22%) CMML patients (data not shown), which suggests that TAK1 deletion is a common event in the pathogenesis of AML and CMML.

## Discussion

In this study, we characterized mice with a conditional deletion of TAK1 in the myeloid lineage. Unexpectedly, ablation of TAK1 in the myeloid lineage resulted in an aggressive and fatal myelomonocytic leukemia with complete penetrance. TAK1^ΔM^ mice have increased numbers of Gr1, CD11b, and Gr1/CD11b double-positive cells in both the bone marrow and spleen of young and diseased mice with a decrease in F4/80^+^ mature macrophages (data not shown). In addition, the Gr1 and CD11b populations show increased proliferation capacity *in vivo*. All TAK1^ΔM^ mice developed early onset of severe splenomegaly as a result of massive infiltration of immature and maturing cells of the myeloid lineage. Histopathologic staining and hematopathologic analysis of peripheral blood, bone marrow, spleen, and other tissues indicated an increased number of immature and maturing monocytic/granulocytic cells in the TAK1^ΔM^ mice, which likely resulted in organ failure as the mice age. Aging TAK1^ΔM^ mice failed to gain weight compared with their TAK1^F/+^ littermates; in addition, they showed signs of morbidity (*i.e.*, ruffled hair, decreased activity, and rapid respiration) and died early, with a median survival duration of 29 weeks. These data are consistent with the clinical observations of myeloproliferative neoplasms and acute myeloid leukemias that generally result in enhanced proliferation and survival of progenitors with an impaired differential potential. Corollary to this postulate, we found that TAK1 deletion is prevalent in a subset of AML patients supporting the importance of TAK1 in both mouse and human leukemia.

Conventional and conditional ablation of the TAK1 gene in the mouse has revealed its essential role for normal physiological development and its involvement in controlling inflammation and anti-apoptotic responses. The pro-survival function of TAK1 is attributed to its well-known ability to directly activate the IKK complex, resulting in the activation of the transcription factor NF-κB, one of the most widely studied regulators of inflammation and survival [33]. Unexpectedly, in the present study, we found that myeloid-specific deletion of TAK1 causes clonal proliferation of myelomonocytic cells in mice. Moreover, unlike conditional deletion of IKKβ with the LysM-Cre line [34], IKKβ floxed mice crossed with the Mx1-Cre line develop neutrophilia, which can be reversed by simultaneous deletion of interleukin (IL)-1R [35] or TNFR1 [36]. In our study, the fatal myelomonocytic leukemia that developed in TAK1^ΔM^ mice was not reversed by the simultaneous deletion of TNFR1; in fact, TNFR1-null TAK1^ΔM^ mice developed splenomegaly and died at 25 to 43 weeks of age. This finding is in sharp contrast to those of previous studies, in which simultaneous deletion of TNFR1 reversed the severe phenotypes observed in TAK1 conditional knockout mice [4], [6], [7]. In addition, NF-κB [37], IKKβ [34], [35], and JNK/p38 [38] knockout mouse models do not develop spontaneous myeloid leukemia. Thus, it is likely that TAK1 loss in the myeloid lineage represents a novel phenotype that cannot be entirely explained by TAK1’s involvement in the NF-κB or stress kinase pathways.

Interestingly, cytogenetic analysis by G-banding of whole bone marrow from TAK1^ΔM^ mice showed multiple chromosomal abnormalities. Chromosomal aberrations were also identified by using array comparative genomic hybridization analysis in liver tumors from conditional deletion of TAK1 in hepatocytes [8]. These two mouse models suggest a correlation between loss of TAK1 and chromosome integrity. Since cells gains aneuploidy characteristics due to aberrant mitotic division [39]–[41], we hypothesized that TAK1 may be regulated during mitosis in addition to its well established role as a MAP3K in response to diverse stimuli. Significantly, we provided evidence for the first time that TAK1 expression and kinase activity are regulated during the mitotic phase of the cell cycle strictly in parallel to well-known mitotic kinases such as Aurora A [42]. Furthermore, unlike ligand-dependent non-proteasomal ubiquitination of TAK1, which consists of Lys63-linked poly-ubiquitination of TAK1 that is necessary for activation of IKK and stress kinases [43]–[45], we showed that TAK1 is ubiquitinated and targeted by the proteasome during mitosis similar to other known regulators of the mitotic phase of the cell cycle [46]. Given that TAK1 is highly activated and regulated during mitosis suggest the possibility of TAK1’s involvement at some molecular level during this process. Consistent with this postulate, a recent finding has shown that knockdown of TAK1 resulted in an increased ploidy index similar to other well-known regulators of the mitotic phase of the cell cycle [31]. Therefore, loss of TAK1 may impact or weaken the mitotic checkpoint and lead to chromosome abnormalities. Our results raise a number of questions for future studies including why TAK1 is activated during mitosis, what its downstream substrates are, and whether it is a substrate for other mitotic kinases as has been reported previously [47], [48]. Nonetheless, the exact mechanism by which TAK1 functions during the mitotic phase of the cell cycle that is critical for maintaining chromosomal integrity [39], [41] requires further investigation.

Given its pro-survival and pro-inflammatory roles, TAK1 has become an attractive target for drug development. Although a number of conditional TAK1 knockout mouse models have provided valuable information about the functional loss of TAK1 *in vivo* that could facilitate the clinical development of systemic TAK1 inhibitors, few studies have investigated the relationship between mutations or loss of the TAK1 gene and cancer in humans. Compagno *et al.* identified two TAK1 mutations in patients with diffuse large B-cell lymphoma, but the significance of these mutations was not further investigated [49]. Furthermore, Liu *et al.* reported that TAK1 deletion is associated with high-grade prostate cancer in humans [50]; Wu *et al.* subsequently found that TAK1 knockdown in prostate stem cells engrafted into mice displayed features of prostatic intraepithelial neoplasia and invasive carcinoma [51]. In addition, Inokuchi *et al.* and Betterman *et al.* investigated specific ablation of TAK1 in the liver of mice and found that TAK1 loss contributes to liver cancer progression [7], [8], findings that were consistent with those of the present study, indicating that TAK1 deficiency has a functional role in cancer development in a cell-context specific manner.

The present study did not unravel the molecular mechanism by which myeloid-specific deletion of TAK1 causes fatal myeloid leukemia in mice. Because it is unlikely that the fatal myeloid leukemia observed in our mouse model can be solely attributed to the canonical TAK1 signaling pathways, further investigations are needed to understand the molecular dynamics at play that lead to the development of this fatal disease. In this instance, a comprehensive study of signature patterns of gene expression in the myeloid compartment of TAK1^ΔM^ mice and their correlation with other CMML and AML mouse models will allow the identification of gene regulatory pathways that lead to CMML in transformation to AML.

In conclusion, we found that TAK1 is a critical regulator of myeloid homeostasis and its loss in the myeloid compartment of mice gives rise to myelomonocytic expansion with rapid progression to an aggressive, fatal myeloid leukemia. Our identification of TAK1 deletion in a subset of AML patients provides strong support for TAK1’s pathobiological role in myeloid leukemogenesis. Further studies investigating the prognostic significance of TAK1 in myeloid neoplasms are warranted. Our findings indicate that TAK1 is an important gatekeeper of myeloid homeostasis and its deletion triggers leukemogenesis.
